# Effect of Heat Treatment on the Microstructure and Properties of a Ti_3_Al Linear Friction Welding Joint

**DOI:** 10.3390/ma12071159

**Published:** 2019-04-10

**Authors:** Xiaohong Li, Jianchao He, Tiancang Zhang, Jun Tao, Ju Li, Yanhua Zhang

**Affiliations:** 1 School of Mechanical Engineering and Automation, Beihang University, Beijing 100191, China; lixhamti06@163.com; 2 Aeronautical Key Laboratory for Welding and Joining Technologies, AVIC Manufacturing Technology Institute, Beijing 100024, China; hjch1985@gmail.com (J.H.); bjsztc@126.com (T.Z.); taojun40@126.com (J.T.); hfutlju@163.com (J.L.)

**Keywords:** intermetallic compound, linear friction welding, microstructure, heat treatment

## Abstract

Heat treatment at different temperatures was carried out on a Ti_3_Al linear friction welding joint. The characteristics and evolution of the microstructure in the weld zone (WZ) and the thermo-mechanically affected zone (TMAZ) of the Ti_3_Al LFW joint were analyzed. Combined with the heat treatment after welding, the effect of the heat treatment temperature on the joint was discussed. The test results indicated that the linear friction welding (LFW) process can accomplish a reliable connection between Ti_3_Al alloys and the joint can avoid defects such as microcracks and voids. The weld zone of the as-welded Ti_3_Al alloy joint was mainly composed of metastable β phase, while the TMAZ was mainly composed of deformed α_2_ phase and metastable β phase. After being heat treated at different temperatures, the WZ of the Ti_3_Al LFW joint exhibited a significantly different microstructure. After heat treatment at 700 °C, dot-like structures precipitated and the joint microhardness increased significantly. Subsequently, the joint microhardness decreases with the increase in temperature. Under heat treatment at temperatures above 850 °C, the formed structure was acicular α_2_ phase and the joint microhardness after heat treatment was lower than that of the as-welded joint.

## 1. Introduction

As a type of Ti-Al series intermetallic compound, Ti_3_Al-based alloys have better high-temperature performance, oxidation resistance, creep resistance, and higher service temperature than ordinary Ti alloys. Compared with the Ni-based alloys, the density of Ti_3_Al-based alloys is lower and about half that of Ni-based alloys, which is beneficial for the reduction in equipment weight. As a high-temperature structural material that can fill the temperature gap between the serving temperatures of Ti alloys and Ni-based superalloys, Ti_3_Al has a good application prospect in the aerospace field, such as to substitute for structural materials with lower strength, to reduce the weight of engines of various vehicles as well as the vehicle’s own weight, and to enhance the specific thrust and efficiency of engines. Owing to the above advantages, Ti_3_Al-based alloys have broad application prospects, since they can meet the urgent needs of future aerospace component structures for light structural materials with high specific strength, high specific modulus, and excellent overall performance [1,2,3,4]. In the application of Ti_3_Al, it is important to solve the jointing problem between the same or different materials that will directly affect its application [5,6].

The main problem in the welding of Ti_3_Al alloys is that the joint has low room-temperature plasticity and is very sensitive to cracks during solidification. The transition from β phase to α phase is one of the main factors affecting the welding of Ti_3_Al. During the welding process of Ti_3_Al alloys, the cooling rate effect on the joint performance plays a decisive role, i.e., as the cooling rate increases, the β phase is cooled rapidly, and the main phase in the microstructure of the welded joint is metastable β phase [7]. The β phase is relatively soft and has a lower fracture toughness, and it is thermodynamically unstable, thus, the transformation from β phase to α_2_ phase will occur in high temperature applications. At a slightly lower cooling rate, a very hard α_2_ (martensite) phase easily forms in the joint and exhibits very high hardness and enhanced brittleness [8,9]. Another problem in the fusion welding of Ti_3_Al is that the thermo-mechanically affected zone (TMAZ) is overheated, and thus, the grains are prone to undergo irreversible coarsening and exhibit significantly increasing brittleness. In addition, hot cracks occur in the joints when a thicker slab is welded. However, the potential advantage of solid-phase welding is to avoid casting structures, gas pores, cracks, deformations, and residual stresses caused by material melting during fusion welding.

As one of the solid-phase welding technologies, weld joints are formed on the contact surface through plastic deformation, surface activation, diffusion, recrystallization, and interaction between rubbing bodies. Linear friction welding (LFW) has a series of advantages, including that the joint is a forged structure, the grain-refined structure is dense, the joint quality is high, there is no dust and weld spatter during the welding process, no filler material and gas protection is needed, and there is less material loss and fewer weld defects [10]. Due to the aforementioned advantages, LFW has been widely used in the welding of carbon steel and stainless steel [11,12,13], aluminum alloys [14,15], titanium alloys [16,17,18,19], and nickel-based alloys [20,21,22], and has become the key technology in the manufacture and repair of Ti-alloy integral discs of aero-engines [23,24]. The LFW process is a thermo-mechanical coupling process that occurs under the action of axial and friction forces, heat generation, phase transformation, and deformation at the welding interface. The phase composition and microstructural characteristics of different materials lead to significant differences in the deformation mechanism and phase transition characteristics in the interface and their vicinity under the thermo-mechanical coupling during LFW. In the LFW process, the main structures of the lamellar-equiaxed dual-state α + β type TC4 Ti alloy in the TMAZ are composed of deformed α phase and acicular martensite, while the weld nugget is composed of martensite [25,26]. In the basket weave structure of TC17 LFW joint, the microstructure in the TMAZ is composed of smaller deformed β grains that formed under the effects of force and temperature duing LFW, and in which residual acicular α phase and metastable β phase are scattered, while the WZ is composed of metastable β phase with fine grains [27,28]. The microstructure of the Ti-Al series of Ti_2_AlNb alloy includes O phase and α phase. In the LFW joint, the microstructure in the TMAZ is metastable β phase, residual O phase, and α phase, where the O phase structure disappears before the α phase. The main phase in the weld nugget zone is metastable β phase with fine grains [29].

Compared to the above materials, the Ti_3_Al alloy has a distinguishable microstructure, since it is mainly composed of β, α_2_, and O phases. The structural and physical properties of β, α_2_, and O phase are significantly different [30]. In the LFW process, the thermo-mechanical action may have great impact on the deformation and phase transition of those three phases. However, the deformation differences of the three phases in the joint and the effect of microstructure variation caused by heat treatment on the performance of the joint have been rarely reported in the literature [31]. In this study, Standard heat treatment of the Ti_3_Al alloy is 980 °C (solution treatment) + 800 °C (aging treatment). LFW was performed on Ti_3_Al alloy, and then the joints were subjected to different heat treatments. The microstructural characteristics of the Ti_3_Al alloy LFW joint and the effect of heat treatment on the microstructure and mechanical properties of the joints were specifically analyzed.

## 2. Materials and Methods

### 2.1. Experimental Materials

**Figure 1:**
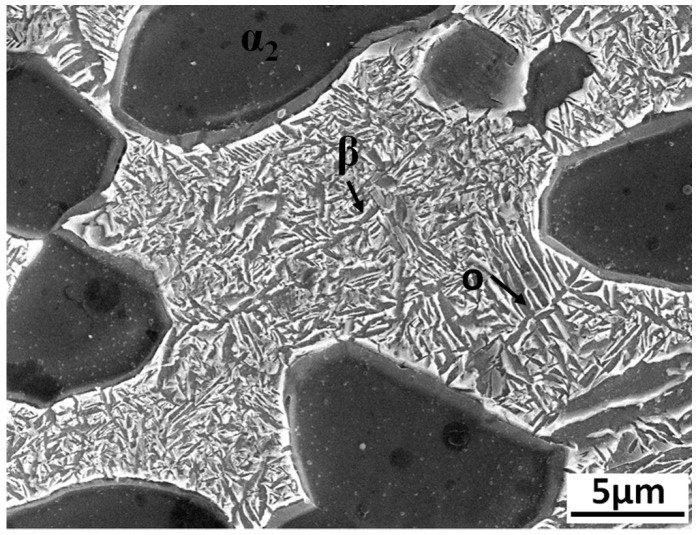
Microstructure of Ti_3_Al-based alloy.

The material used in experiment was Ti_3_Al-based alloy (Ti-23Al-17Nb (at%)). As it can be seen from Figure 1, the original structure of the alloy is a mixture of α_2_, O, and β phases, where the matrix phase is α_2_ with an approximate size of 15 μm. Numerous acicular O with a length of 10 μm and alternate β phases are distributed across the equiaxed α_2_ phase matrix and its chemical composition can be seen in Table 1.

### 2.2. Experimental Methods

**Figure 2:**
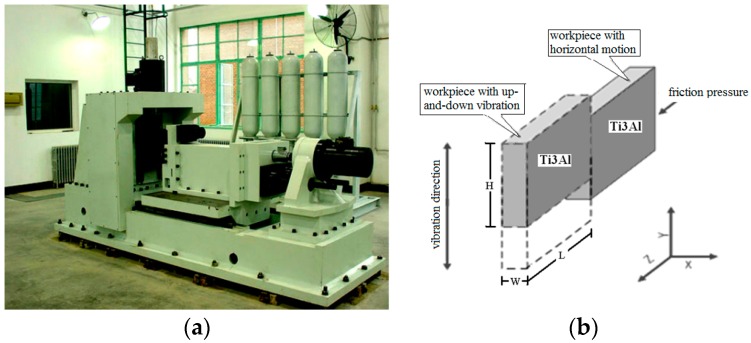
(**a**) The LFW-20T equipment; (**b**) Basic mechanism of the LFW process.

The LFW test was carried out on an LFW-20T equipment, self-developed by AVIC Manufacturing Technology Institute (Beijing, China). The equipment is shown in Figure 2a and the welding principle of the LFW process is shown in Figure 2b. One specimen of a pair to be welded was held by a clamp with a reciprocating motion and the other was held by a clamp having a horizontal motion. During welding, one specimen started to reciprocate with high frequency and small amplitude under the action of the exciting force, while the other gradually moved toward the reciprocating specimen under the action of thrust. Once the specimens come into contact, the convex portions of the interface rub each other. As the frictional pressure increased, the actual contact area increased, and then the frictional force increased rapidly. Accordingly, the interface temperature increased and the friction interface was gradually covered by a layer of high-temperature visco-plastic metal. Under the action of upset pressure, the metal in the welding zone underwent plastic flow and the extruded metal formed a flash. The metal components on the two sides were firmly welded together through mutual diffusion and recrystallization of the metal in the welding zone, and the entire welding process was completed.

The parameters of the Ti_3_Al LFW process are listed in Table 2.

**Figure 3:**
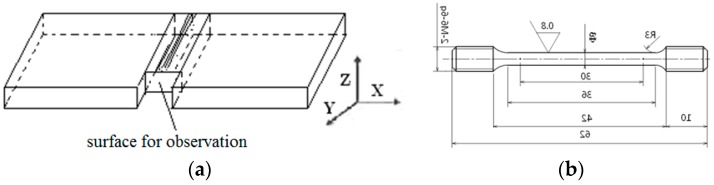
(**a**) Schematic diagram of the intercepting process for the metallographic specimen; (**b**) Tensile specimen dimensions.

The cooling rate is very fast after LFW, and the joint is very narrow with severe plastic deformation. So the range from 700 °C–900 °C was used to research the evolution of microstructure and Properties of the Ti_3_Al joint. After the LFW process, the joints were heated in vacuum at 700 °C, 750 °C, 800 °C, 850 °C, and 900 °C for four hours and were cooled in a furnace. The specimen for microstructure characterization was taken along the direction perpendicular to the welded surface, and then the sample was ground and polished. Aqueous solutions of hydrofluoric acid and nitric acid were adopted successively to etch the sample. Subsequently, the sample was observed using a Zeiss field-emission scanning electron microscope (Jena, Germany) at the test center of the China Aviation Manufacturing Technology Research Institute. The specimen for microhardness measurement was subjected to several processes, including inlaying, polishing, and etching. The microhardness characterization was performed on an Tukon2500 microhardness tester (Wolpert Wilson, IL, USA), where the microhardness of an as-welded joint and a welded joint after heat treatment was measured at a load of 0.2 kg for 15 s. Based on the distance from the center of the joint, at least five points were selected to measure the microhardness of each zone, among the base metal (BM), the TMAZ, and the weld zone (WZ) of the Ti_3_Al joint. The point-to-point spacing was greater than 0.2 mm. The position for the microstructure and microhardness measurements is shown in Figure 3a. A tensile test piece was prepared according to the HB5143-96 standard, in order to measure the tensile properties of the joints at room-temperature, as shown in Figure 3b. The center line of the joint remained at the center of the tensile test sample and three test pieces were taken from the specimens under different heat treatment conditions.

## 3. Results and Discussions

### 3.1. Microstructural Characteristics of the As-Welded Joint

**Figure 4:**
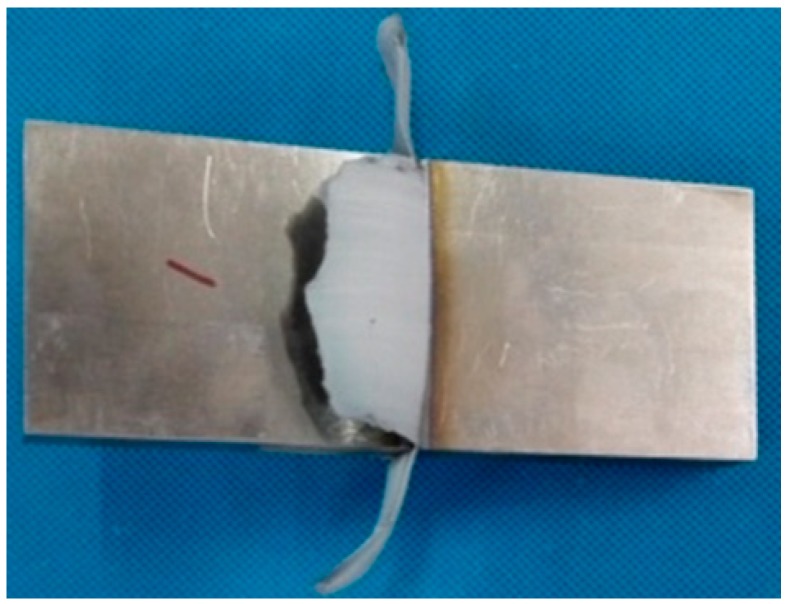
Morphology of the Ti_3_Al joint after LFW.

**Figure 5:**
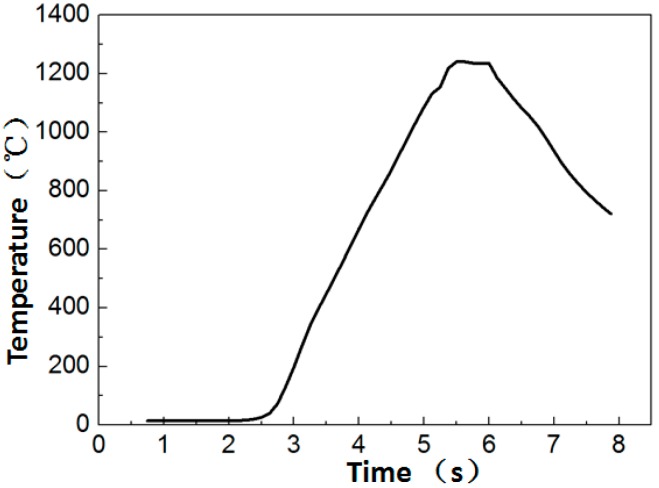
Temperature curve measured during Ti_3_Al LFW.

The morphology of Ti_3_Al after LFW can be observed in Figure 4. At the welding interface, under the action of pressure and friction forces, the interfacial temperature increased, then the Ti_3_Al at the interface reached its molten state, and thus Ti_3_Al was extruded and formed a flash. After being cooled in air, the flash was grayish white with the same direction. During the LFW process, different materials have flashes with different morphologies, which is highly related to the material physical properties. More specifically, it is easier to form flashes that are extruded as whole bodies from materials with high plasticity and fluidity. As for materials with poor plasticity, during extrusion, the flash separates and curls toward both sides. The morphology of the flash formed by LFW on the Ti_3_Al alloy is similar to that of ordinary Ti-based alloys, such as TC4, TC11, and TC17, and exhibits integral extrusion (Figure 4). In the linear friction welding process of Ti_3_Al, the welding temperature can reach over 1200 °C, as illustrated in Figure 5.

**Figure 6:**
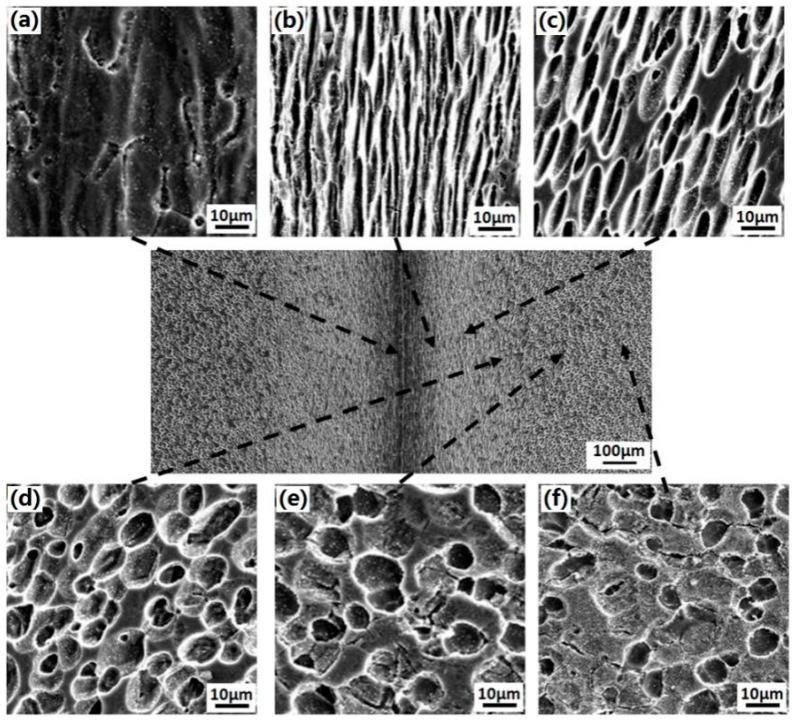
Microstructures in different zones of the as-welded Ti_3_Al LFW joint: (**a**) in WZ, (**b**) in TMAZ close to WZ, (**c**) in TMAZ, (**d**) in TMAZ close to BM, (**e**) in BM close to TMAZ, and (**f**) in the BM.

In Figure 6, it can be seen that there were no defects such as voids, cracks, and unwelded regions at the welded interface of the Ti_3_Al LFW joint. The Ti_3_Al LFW joint can be divided into three zones: the BM zone, the TMAZ, and the WZ. The width of the WZ was about 80 μm and the entire width of the joint was 700 μm. During the LFW process of Ti_3_Al, heat was generated at the contact surface under the action of pressure and vibration friction, and then was conducted towards the base metal, which formed a high-to-low temperature gradient from the contact surface to the base material. Meanwhile, under the action of friction pressure, the metal at the interface flowed and extruded from the interface, resulting in a greater deformation of the microstructure. Therefore, the Ti_3_Al LFW joint was characterized by a microstructure with gradient variation formed at different temperatures and deformation degrees. The microstructure in the different zones of the joint was quite different. In the BM zone, the morphology, distribution, and quantity of the α_2_, O, and β phases demonstrated had not obvious change, as shown in Figure 6a. In the TMAZ near the side of the BM zone, the lamellar O and β phases deformed and after cooling formed a metastable structure, while the morphology of the α_2_ phase was not significantly deformed, as shown in Figure 6e. At regions closer to the joint center, the α_2_ phase began to deform and the metastable portion increased as the metal was elongated along the direction of the flow, as shown in Figure 6d. In the TMAZ, close to the side of WZ the α_2_ phase increased severely and the length-width ratio increased, almost parallel to the joint center, as shown in Figure 6c. At regions closer to the central part of joint’s interface, the α_2_ phase further reduced, as shown in Figure 6b. In the WZ, the α_2_, O, and β phases all transformed to the metastable β phase, except a small amount of deformed a_2_ phase, as shown in Figure 6a.

### 3.2. Effects of Heat Treatment on Joint Microstructure

**Figure 7:**
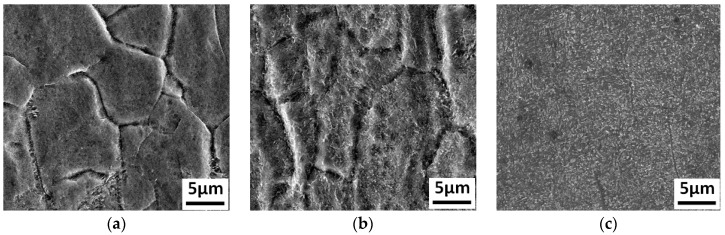

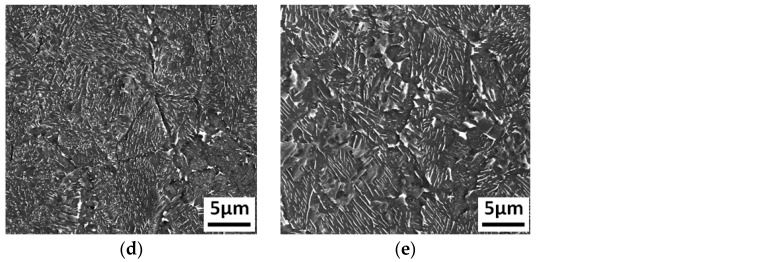
The Ti_3_Al LFW joint microstructures in the WZ after heat treatments at different temperatures: (**a**) after heat treatment at 700 °C, (**b**) after heat treatment at 750 °C, (**c**) after heat treatment at 800 °C, (**d**) after heat treatment at 850 °C, and (**e**) after heat treatment at 900 °C.

The Ti_3_Al LFW joints were heat treated at 700 °C, 750 °C, 800 °C, 850 °C, and 900 °C, respectively, and the corresponding microstructures after heat treatment are shown in Figure 7. After being heat treated at 700 °C, the central zone of the joint was composed of small equiaxed grains with a size of around 10 μm. There was a wider grain boundary and dot-like structures less than 1 μm precipitated inside the grains, as shown in Figure 7a. Mount of the dot-like structures (white color point) appeared in the grain and grain boundary reduced in the width, as shown in Figure 7b,c. Under higher temperature, the dot-like structures inside the equiaxed grains began to grow and transform to short acicular structures at 850 °C, as shown in Figure 7a. When the temperature reached 900 °C, the size of the short acicular structures inside the equiaxed grains was close to 3 μm, and exhibited a trend of becoming lamellar, as shown in Figure 7e.

**Figure 8:**
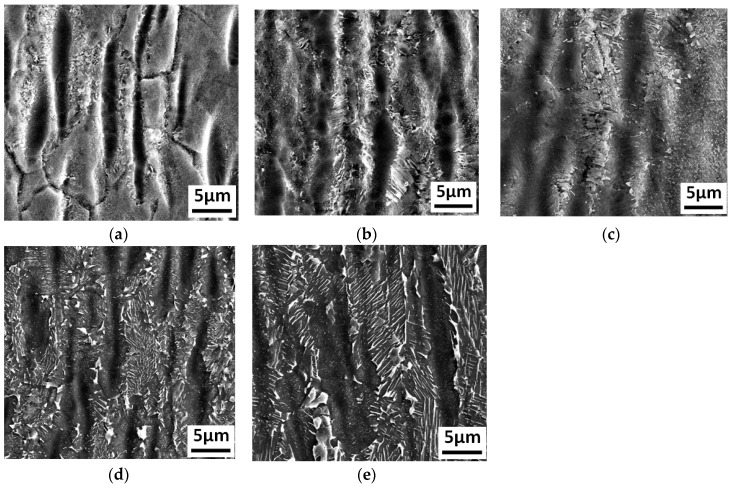
The Ti_3_Al LFW joint microstructures in the TMAZ after heat treatments at different temperatures: (**a**) after heat treatment at 700 °C, (**b**) after heat treatment at 750 °C, (**c**) after heat treatment at 800 °C, (**d**) after heat treatment at 850 °C, and (**e**) after heat treatment at 900 °C.

The TMAZ of the as-welded joint was mainly composed of deformed α_2_ phase and metastable structures. After heat treatment at 700 °C, the morphology of the residual deformed α_2_ phase did not change significantly compared to that in the as-welded joint. The dot-like structures precipitated between α_2_ phases, while short acicular structures appeared locally, as shown in Figure 8a. As the temperature increased, the size of the dot-like structures increased, while the size of the α_2_ phase decreased, which was consistent with the variation in the dot-like structures inside the equiaxed grains at the center of the joint, as shown in Figure 8b. More specifically, at 800 °C, the morphology of the dot-like structure began to transform into short acicular structure, as shown in Figure 8c. It began to exhibit lamellar structure and the size of the α_2_ phase decreased further at 850 °C, as shown in Figure 8d. The average length of lamellar structures is up to 5 μm, as shown in Figure 8e.

**Figure 9:**
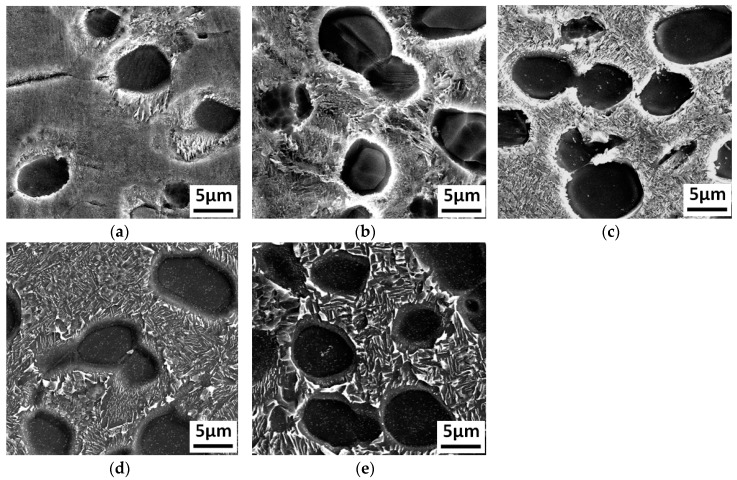
The Ti_3_Al LFW joint microstructures in the BM zone after heat treatments at different temperatures: (**a**) after heat treatment at 700 °C, (**b**) after heat treatment at 750 °C, (**c**) after heat treatment at 800 °C, (**d**) after heat treatment at 850 °C, and (**e**) after heat treatment at 900 °C.

The microstructure variation in the TMAZ close to the side of the base metal was different than that in the other two zones. The morphology of the α_2_ phase did not change significantly with temperature, while the heat treatment led to larger impact on the microstructures between the α_2_ phases. At 700 °C, the microstructures between the α_2_ phases exhibited dot distribution, as shown in Figure 9a. After heat treatment at 750 °C, the dot-like structures transformed to acicular structures, and then grew rapidly with the increase in temperature, as shown in Figure 9b. At 800 °C, the length of the acicular structures α_2_ reached 3 μm but still exhibited a narrow shape, as shown in Figure 9c. At 850 °C, the acicular structures α_2_ is larger than that in the 800 °C sample, as shown in Figure 9d. After heat treatment at 900 °C, the length reached approximately 5 μm, as shown in Figure 9e

In the LFW process, under the combined action of axial and friction forces, the contact surface of the Ti_3_Al alloy undergoes friction, deformation, heat generation, and extrusion of the interface metal. Therefore, the microstructure of the Ti_3_Al LFW joint is affected by welding process parameters, such as friction pressure, welding amplitude, welding frequency, and cooling rate. The welding temperature of Ti-based alloys during LFW can reach temperatures above 1200 °C. Since the cooling rate after welding is relatively fast, martensite structures can form in TC4 titanium alloys, while metastable β phase can form in Ti17 titanium alloys and near-b titanium alloy [19,32]. From the phase diagram of Ti_3_Al-based alloys [33], it can be seen that the Ti_3_Al material has three phase regions: the β + O phase region, the α_2_ + β + O phase region, and the β phase region. The microstructures of the as-welded Ti_3_Al alloy LFW joint indicated that the joint forming between the base material and the interface contains different microstructures, including an α_2_ + O + β three-phase region, an α_2_ + metastable β dual-phase region, and a metastable β phase region. In the welding process and under the action of friction pressure, deformation occurs in the aforementioned four phase regions ranging from the base metal to the weld interface. 

The degree of deformation is related to the amplitude and extent of the softening and flow of the Ti_3_Al alloy. The closer to the weld interface, the more severe the deformation. In the welding process, two main transitions occur: O phase → β phase and α_2_ phase → β phase. In the weld zone, the temperature, which is over 1200 °C, leads to O phase → β and α_2_ phase → β phase transitions, but the LFW process is short, so the α_2_ phase → β phase transition is incomplete. Thus, there is a small amount of deformed α_2_ phase and the extrusion of more softened alloy, which makes the weld spacing narrower after the upsetting force formation process of LFW. The zone is mainly composed of β phase and a small amount of deformed α_2_ phase. Due to the higher cooling rate of LFW, the disordered β phase transits into an ordered β phase, while the α_2_ phase remains. As the distance from the weld zone increases, the maximum welding temperature decreases, so the corresponding temperature range is located in the α_2_ + β dual-phase region. Within this temperature region, the O phase transforms to β phase, while the partial α_2_ phase transforms to β phase. During the cooling process, the residual deformed α_2_ phase can remain until room temperature, therefore the α_2_ portion in the WZ is higher than that in the TMAZ close to near the WZ. For the TMAZ close to the BM, the welding temperature is relatively low so the high temperature region is located at the α_2_ + β + O phase region and the β + O phase region. Within this temperature region, the α_2_ phase is relatively stable and basically does not transform into β phase. Due to the fact that the O phase has poor stability, the transition of O phase → β phase occurs easily during the welding process. The higher the temperature, the more complete the transition and the lesser the O phase. Therefore, in the TMAZ near the base metal of the as-welded joint under thermo-mechanical action, the O, β, and α_2_ phases in the deformed microstructure do not transform to β phase. The difference in the relationship between temperature and stress/strain in the different regions of the material during the LFW process has a great and large-area impact on the microstructure (morphology, size, and volume fraction of the β, α_2_, and O phases) at the different joint regions. After heat treatment, the metastable β phase decomposes into α_2_, O, and β phases. The significant differences in the microstructure of each zone of the as-welded joint are responsible for the large differences in the microstructure of each zone of the joint after heat treatment. The more obvious impact is that of the different heat treatment temperatures on the size and morphology of the α_2_ phase formed by the decomposition of the metastable β phase.

### 3.3. Effects of Heat Treatment on Joint Microhardness

**Figure 10:**
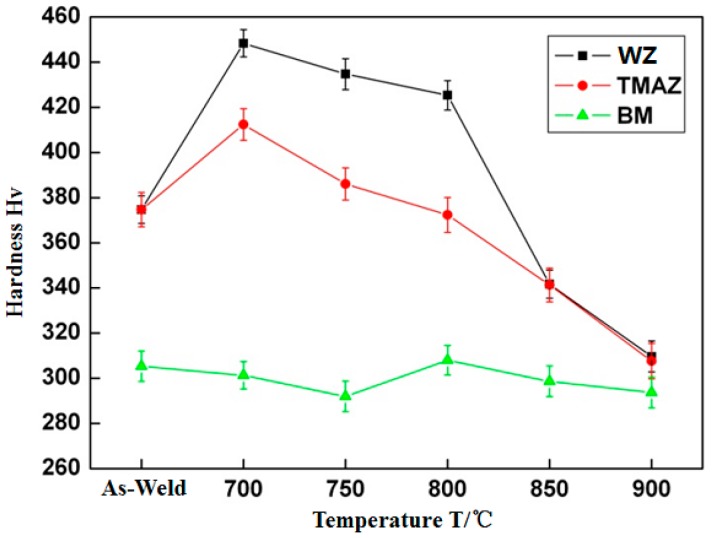
Microhardness of as-welded and heat-treated joints.

The microhardness tests were carried out on as-welded Ti_3_Al LFW joints and joints after different heat treatments. The variation of microhardness with temperature is shown in Figure 10. In the as-weld condition, the microhardness of the Ti_3_Al weld zone and the TMAZ was similar. It reached about 375 Hv, much higher than the microhardness of the base metal (300 Hv). After heat treatment at 700 °C, the microhardness in the weld zone increased rapidly to 450 Hv, while that in the TMAZ also increased to 425 Hv, and in the base metal did not change significantly. As the heat treatment temperature increased, the joint microhardness in the WZ and the TMAZ gradually decreased. After heat treatment at 800 °C, the joint microhardness in the TMAZ was close to that of the as-welded joint. When the heat treatment temperature reached 850 °C, the microhardness in the WZ and the TMAZ was much lower than that of the corresponding zones of the as-welded joint, which was reduced to approximately 340 Hv. When the heat treatment temperature was raised to 900 °C, the joint microhardness in the WZ and the TMAZ was close to that of the base metal, which was about 300 Hv. The LFW process is a non-uniform deformation process in which the temperature varies from high to low and the stress/strain also vary from high to low when the location changes from the base metal region to the weld zone. In the secondary process, accompanied with processing-hardening, phase transition, and dynamic recrystallization, there are variations in the quantity, size, and morphology of the α_2_, O, and β phases. For instance, the predominant action on the zone close to the base metal is processing-hardening, while the phase transition is predominant in the weld zone. As a result, the microhardness of the TMAZ and the WZ of the Ti_3_Al joint increases. The heat treatment on the Ti_3_Al LFW joint is equivalent to the aging treatment on the Ti_3_Al after rapid cooling and with the deformation in the O + β, α_2_ + β + O, α_2_ + β, and β phase regions. In the treatment, the α_2_ phase precipitates from the metastable β matrix, which produces a strengthening effect and increases the microhardness. However, when the heat treatment temperature is too high, the α phase grows, decreasing the microhardness and reducing the strengthening effect.

### 3.4. Effects of Heat Treatment on Joint Tensile Properties

**Figure 11:**
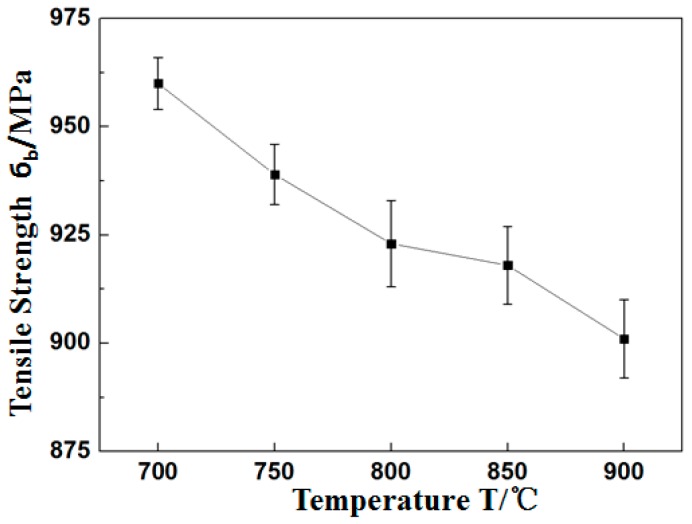
Tensile strength results with heat treatment from 700 °C to 900 °C.

**Figure 12:**
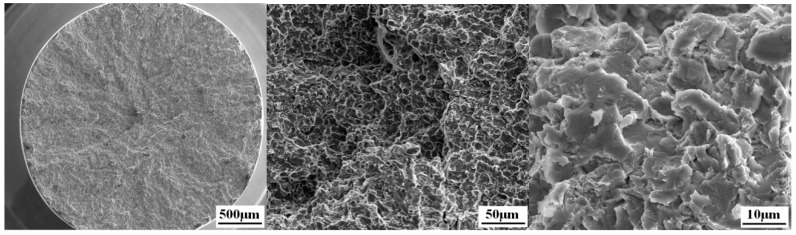
Tensile fracture morphology of the Ti_3_Al LFW joint at room temperature.

After heat treatment at 700 °C, 750 °C, 800 °C, 850 °C, and 900 °C, the tensile strength (б_b_) of the Ti_3_Al LFW joints at room temperature was 960 ± 6 MPa, 939 ± 7 MPa, 923 ± 10 MPa, 918 ± 9 MPa, and 901 ± 9 MPa, respectively, and tensile strength (б_b_) of joints decreased with the temperature of heat treatment from 700 °C to 900 °C, as show in Figure 11. All fractures occurred in the BM zone of Ti_3_Al and there was a certain bottleneck shrinking of about 10%. As it can be seen in Figure 12, the macro-fracture was a typical cup-shaped fracture and was composed of a fibrous zone and a shear lip zone. The shear lip zone was more apparent, while the fibrous area exhibited a shallow dimple shape. After being subjected to heat treatment, the microhardness of the WZ and the TMAZ of the Ti_3_Al LFW joint was higher than that of the base metal, which was one of the reasons for the tensile fracture at the base metal region at room temperature.

## 4. Conclusions

(1) The microstructure in the central WZ of Ti_3_Al LFW joint is metastable β phase. The microstructure in the TMAZ is deformed α_2_ phase and metastable β phase. As the position becomes closer to the TMAZ near the side of the WZ, the α_2_ phase gradually decreases, the degree of deformation increases, and the metastable β phase increases.
(2) After being subjected to heat treatment at different temperatures, the microstructure in the WZ of the joint is quite different. At 700 °C, dot-like structures precipitate and their size is less than 1 μm. However, when the heat treatment temperature is above 850 °C, the formed structure is acicular α_2_ phase with a size of approximately 1 μm, which grows as the heat treatment temperature increases.
(3) The microhardness of the Ti_3_Al LFW joint after welding is higher than that of the base metal. Heat treatment at 700 °C can significantly enhance the joint microhardness, which then decreases with the increasing heat treatment temperature. After heat treatment at 850 °C, the joint microhardness is lower than that of the as-welded joint. The microhardness of the WZ and the TMAZ is higher than that of the BM zone in the selected heat treatment range.

## Figures and Tables

**Table 1:** Chemical composition of Ti_3_Al-based alloy titanium alloy (wt%).

Al	Nb	Ti
12.44	31.71	Bal.

**Table 2:** Welding process parameters.

	Amplitude (mm)	Frequency (Hz)	Friction Pressure (T)
Optimized welding process parameters	3	40	4
